# Responses of Soil Bacteria Communities to Organic Material Application and Their Antagonistic Activity against *Diaporthe destruens* Causing Sweet Potato Foot Rot Disease

**DOI:** 10.1264/jsme2.ME25011

**Published:** 2025-09-06

**Authors:** Zin Mar Soe, Masao Sakai, Sakura Kihara, Daisuke Fukahori, Masayuki Nakamura, Daisuke Ueno, Jun-ichi Sakagami, Makoto Ikenaga

**Affiliations:** 1 Graduate School of Agriculture, Forestry and Fisheries, Kagoshima University, 1–21–24, Korimoto, Kagoshima, 890–0065, Japan; 2 Research Field in Agriculture, Agriculture Fisheries and Veterinary Medicine Area, Kagoshima University, 1–21–24, Korimoto, Kagoshima, 890–0065, Japan; 3 The United Graduate School of Agricultural Sciences, Kagoshima University, 1–21–24, Korimoto, Kagoshima, 890–0065, Japan; 4 Faculty of Agriculture, Kagoshima University, 1–21–24, Korimoto, Kagoshima, 890–0065, Japan; 5 Faculty of Agriculture, Saga University, 1 Honjomachi, Saga, 840–8502, Japan

**Keywords:** *Diaporthe destruens*, indigenous soil bacteria, organic materials, *Kitasatospora*, antagonistic ability

## Abstract

Sweet potato foot rot disease caused by *Diaporthe destruens* (formerly *Plenodomus destruens*) severely affects the yield and quality of sweet potatoes. To gain basic knowledge on regulating the pathogen using indigenous soil bacteria, the following organic materials were applied to potted soils collected from a sweet potato field contaminated with *D. destruens*: Kuroihitomi (compost made from shochu waste and chicken manure), Soil–fine (material made by adsorbing shochu waste on rice bran), and rice bran. Soil samples were periodically collected during an incubation for bacterial colony counts and a community ana­lysis using a meta 16S amplicon ana­lysis. The number of bacterial colonies was significantly higher with the Soil–fine and rice bran treatments and slightly higher with the Kuroihitomi treatment than with a chemical fertilizer as the control, and then gradually decreased over time. An amplicon ana­lysis showed that the Soil–fine and rice bran treatments increased the relative abundance of *Streptomycetaceae* and *Micrococcaceae* belonging to *Actinobacteria* and *Burkholderiaceae* belonging to *Beta–proteobacteria*. The Kuroihitomi treatment also increased the relative abundance of *Streptomycetaceae*. The dominant amplicon sequencing variant (ASV) sequences among these families were affiliated with the genera *Kitasatospora*, *Arthrobacter*, and *Paraburkholderia*. Bacteria with sequences identical to these ASVs were isolated from the incubated soils using selective media for dual culture assays. Bacterial isolates in a cluster of *Kitasatospora* exhibited antagonistic activity against *D. destruens*. The present results suggest that combining organic materials with antagonistic bacteria may be an effective approach to regulating the growth of *D. destruens*.

Soil is a highly complex ecosystem that represents the most diverse habitat for microorganisms on Earth, providing an ideal reservoir for microorganisms (Torsvik and Øvreås, 2002; Dischinger *et al.*, 2009). Soil microorganisms are major drivers of soil ecosystem functions, such as the decomposition of organic matter, nutrient cycling, and resistance to soil–borne diseases (Mendes *et al.*, 2011; Zhong *et al.*, 2020). Soil organisms respond to changes caused by crop cultivation methods (Drury *et al.*, 1991), fertilizer and pesticide use (Fauci and Dick, 1994), and the manipulation of organic additives (Franzluebbers *et al.*, 1995). The application of organic materials, such as livestock manure, crop residue, compost, green manure, agro–industrial waste, and their combinations, has been shown to improve soil biological properties, such as microbial biomass development and soil metabolic activity (García-Gil *et al.*, 2000), as well as the richness and diversity of microbial communities (Hiddink *et al.*, 2005; Hartmann *et al.*, 2015).

The beneficial effects of the application of organic materials may directly control soil–borne pathogens by releasing fungal toxic compounds (Blok *et al.*, 2000; Tenuta and Lazarovits, 2004; Larkin and Griffin, 2007) or indirectly by promoting the development of a suppressive microbiome (Hoitink and Boehm, 1999; Bonanomi *et al.*, 2018). Previous studies reported that the application of rice bran (RB) markedly changed the bacterial composition and led to an increase in the relative abundance of Gram–positive bacteria, such as *Streptomyces* spp. The majority of isolated *Streptomyces* spp. exhibited antagonistic activity to control potato common scab (Tomihama *et al.*, 2016). The application of RB was also found to suppress the occurrence of sweet potato soil rot disease caused by *Streptomyces ipomoea* (Kanaiso and Yonemoto, 2003) and exhibited nematicidal activity against root–knot nematodes (Taba *et al.*, 2008). Our research group revealed that shochu post–distillation slurry, an ingredient of Kuroihitomi (KH), affected the bacterial composition of soil and led to the largest increase in the relative abundance of *Priesta aryabhattai* (formerly *Bacillus aryabhattai*), which exhibited antagonistic activity against pathogenic *Streptomyces scabiei* causing sweet potato common scab (Takegoshi *et al.*, 2020, 2022).

Sweet potato foot rot disease caused by *Diaporthe destruens* leads to considerable economic losses by significantly reducing sweet potato yields. Sweet potatoes are an important commercial crop with extensive uses as a vegetable, starch source, in confectionery, processed foods, feed, and distilled liquor manufacturing. The occurrence of foot rot in Japan was confirmed in Okinawa prefecture in November 2018, Kagoshima prefecture in December 2018, and Miyazaki prefecture in January 2019 (Kobayashi, 2019). In recent years, outbreaks of sweet potato foot rot caused by *D. destruens* have also been documented in Taiwan (Huang *et al.*, 2012), China (Gai *et al.*, 2016), and South Korea (Paul *et al.*, 2019). In pathogen–infected fields, small black necrotic lesions that disrupt water and nutrient uptake appear along the stems of sweet potatoes. As the disease progresses, infected branches become shriveled and dry, and stems die and turn black due to girdling. Severe infection eventually reaches the storage root, causing it to rot in the soil before harvesting (Gai *et al.*, 2016).

Strategies to protect against *D. destruens* include crop rotation, planting with healthy seeds, using resistant cultivars, and applying agrochemicals (Lopes and Silva, 1993; Reddy, 2015). Sweet potato plant diseases may be effectively controlled by proper sanitation procedures (Lopes *et al.*, 1994; Clark and Moyer, 2013). Shima *et al.* (2024) reported that the combination of countermeasures, resistant varieties, the residual treatment of decayed potatoes after harvesting, crop rotation, and drainage measures need to be implemented in order to reduce severe infection by *D. destruens* as a priority. The newly released cultivar “Konaishin”, developed for starch use and high yield, exhibits high resistance to foot rot disease (Kobayashi, 2020). However, the “Koganesangan” sweet potato variety, which has limited disease resistance, is popular for its delicious flavor and sweetness, making it a favorite in the production of traditional Japanese Imo shochu (Sakai *et al.*, 1967; Kamiwatari *et al.*, 2006; Kamiwatari and Setoguchi, 2011). There are currently no agrochemicals that effectively control the disease (Kobayashi, 2019). Moreover, the heavy use of fungicides is not recommended in sweet potato production due to Japanese food safety regulations. Additionally, the high use of fungicides may lead to the development of fungicide resistance in *D. destruens* (Batzer and Mueller, 2020). Therefore, there is an urgent need to find alternative, environmentally friendly approaches that effectively control sweet potato foot rot disease in Japan.

Microbiological approaches to investigate the effects of organic material applications against pathogen growth by responsive soil bacteria have not been extensively studied in sweet potato cultivation. Although this microbiological approach may not be as effective as pesticides, it is an important method for maintaining a sustainable agricultural system that has less of a negative impact on soil bacteria and the surrounding environment. Previous findings showed that the application of organic materials may increase the activity of indigenous microbiomes, which has been associated with the suppression of various plant pathogens in soil (Li *et al.*, 2019). This leads to the phenomenon of “disease suppressiveness”, referring to the natural ability of the soil to suppress disease development even in the presence of actively growing plant pathogens. This property is attributed to the abundance of several taxa with antagonistic activity against plant pathogens, mediated by the production of antibiotics and antifungal compounds, hydrolytic enzyme activity, and competition for available nutrients (Corato, 2020).

Based on the concerns described above, different organic materials were applied to potted soil collected from a sweet potato field contaminated with *D. destruens* and the responses of the soil bacterial community over the incubation periods were exami­ned. The dominant bacteria, particularly those that responded in the soil after the application of organic materials, were isolated and evaluated for their antagonistic activity against *D. destruens* causing sweet potato foot rot disease, with the aim of obtaining basic knowledge that will contribute to the regulation of this pathogen by indigenous soil bacteria.

## Materials and Methods

### Preparation of soil pots and periodical soil sampling during the incubation

Soil samples were collected from a sweet potato field in Chiran–cho Kori, Minami–Kyushu city, Kagoshima Prefecture, Japan (31°32′03″N 130°43′48″E) before fertilizing and planting. *D. destruens* was spread on this soil. The soil was collected and passed through a 1–mm mesh sieve to remove plant debris, and prepared soil was regarded as 0–time soil. Soil pH and moisture content were measured in duplicate before the application of organic materials.

Three organic materials were used in this study: KH, Soil–fine (SF), and RB. KH is a compost made from the post–distillation slurry of shochu and chicken manure and is manufactured by Technomax Minami–Nihon. SF was made by absorbing the post–distillation slurry of shochu onto RB, manufactured by Katakura &‍ ‍Co–op Agri. Three treatments (KH, SF, and RB) and chemical fertilizer (CF) as a control were prepared in a 1/5,000 Wagner pot containing 3‍ ‍kg of soil. The N:P:K ratio for all treatments was adjusted to 15:30:30. Each soil pot contained 10‍ ‍g of KH and 0.158‍ ‍g of KCl for the KH treatment, 10‍ ‍g of SF and 0.656‍ ‍g of Ca_3_(PO_4_)_2_ for the SF treatment, 13‍ ‍g of RB and 0.723‍ ‍g of KCl for the RB treatment, and 1.415‍ ‍g of (NH_4_)_2_SO_4_, 1.311‍ ‍g of Ca_3_(PO_4_)_2_, and 0.950‍ ‍g of KCl for the CF treatment.

These pots were left open and incubated at 30°C. During the incubation, deionized water was sprayed and supplied every few days to maintain moisture based on weight loss. Soil samples were periodically collected five times from each treatment at 1‍ ‍week, 2‍ ‍weeks, 1 month, 2 months, and 4 months after the incubation using a soil core sampler. Soil moisture content was measured in duplicate. All collected soil samples were used in subsequent experiments.

### Enumeration of bacterial colonies using the culture–dependent cultivation method

The total number of bacterial colonies in soil samples was counted using the agar culture method. Soil samples from each treatment were serially diluted, and aliquots of the suspension were spread on 1/10 tryptic soy agar (TSA) (Martin, 1975) in triplicate. Kabicidin (Fujifilm) was added to prevent the formation of fungal colonies. After a 2–week incubation at 30°C, all bacterial colonies were counted, and colony–forming units (CFU) g^–1^ of dry soil were calculated.

### Investigation of DNA extraction efficacy and PCR amplification

Since the soil used in the present study was Andosol according to the FAO soil classification, the efficiency of DNA recovery from soil was investigated in advance by adding casein sodium. The First DNA Spin Kit for Soil (MP Biomedicals) was used for the extraction using 0–time soil, modified by adding different concentrations of casein sodium. Different amounts of casein sodium (5, 10, 15, 20, 25, 30, 35, and 40‍ ‍mg) per extraction tube were individually dissolved in sodium phosphate buffer supplied with the kit. A casein sodium–free tube (0‍ ‍mg) was also prepared as a control. Total DNA extraction from 0.5‍ ‍g soil was performed according to the manufacturer’s protocols with the addition of the phenol/chloroform/isoamyl alcohol (25:24:1) (PCI) and then chloroform/isoamyl alcohol (24:1) (CIA) treatments. During the extraction, the amount of reagent was adjusted appropriately at each step based on the amount of recovery solutions containing DNA. The concentration of extracted DNA with different amounts of casein sodium was measured with an Invitrogen Qubit 3.0 fluorometer (Thermo Scientific).

Amplification of the bacterial 16S rRNA gene was performed on extracted DNA using the primers 341f (*E. coli* positions 341–357; 5′–CCTACGGGNGGCWGCAG–3′) and 805r (*E. coli* positions 805–785; 5′–GACTACHVGGGTATCTAATCC–3′) (Klindworth *et al.*, 2012), which are commonly used for a meta 16S rRNA amplicon sequence ana­lysis targeting the bacterial V3 and V4 regions. The PCR reaction was conducted in a final volume of 25‍ ‍μL, containing 12.5‍ ‍μL EmeraldAmp MAX PCR Master MixPremix (Takara), 1‍ ‍μL of both primers (20 pmol μL^–1^ each), 10‍ ‍ng of extracted DNA, and sterilized ultra–pure water. The PCR program was as follows: initial denaturation at 94°C for 3‍ ‍min, then 30 cycles consisting of denaturation at 94°C for 1‍ ‍min, annealing at 54°C for 1‍ ‍min, and extension at 72°C for 1.5‍ ‍min, followed by a final extension at 72°C for 8‍ ‍min. Aliquots of PCR products were separated by electrophoresis in a 1.5% agarose gel and amplification products were visualized by staining with ethidium bromide under UV illumination.

### Community structure of soil bacteria by the meta 16S amplicon ana­lysis

The community ana­lysis of soil bacteria in the four different treatments was performed using a paired–end method with MiSeq (Illumina), targeting the V3 and V4 regions of bacterial 16S genes. DNA was extracted from 0.5‍ ‍g of soil in triplicate with the addition of appropriate amounts of casein sodium. The concentrations of extracted DNA were measured using a Qubit 3.0 fluorometer. PCR products were generated using a set of primers, 341f and 805r, with adaptor sequences, and index sequences were subsequently attached to the products through amplification. The index sequences attached to the 5′ ends of the forward and reverse primers were as follows: 5′–ACACTCTTTCCCTACACGACGCTCTTCCGATCT–3′ for 341f and 5′–GTGACTGGAGTTCAGACGTGTGCTCTTCCGATCT–3′ for 805r.

The Quantitative Insights into Microbial Ecology (QIIME 2) pipeline (Caporaso *et al.*, 2010) was used to remove low–quality and chimeric sequences. The sequences that passed preprocessing were obtained as amplicon sequence variants (ASVs) at 97% similarity, together with the representative sequences. The procedure described above was performed by Bioengineering Lab. (http://www.gikenbio.com/). The closest relatives of the ASV sequences were identified using EZBioCloud (https://www.ezbiocloud.net). All bacterial 16S rRNA gene sequences obtained from the meta 16S amplicon ana­lysis were registered in Genebank under accession number PRJNA1112978.

### Isolation of bacteria whose relative abundance was increased by the application of organic materials

To isolate bacteria whose relative abundance was increased by the application of organic materials, the KH and SF treatment soil pots were prepared again and incubated at 30°C for 2‍ ‍weeks. Soil samples were collected, serially diluted, and aliquots of the dilutions were then spread on three different media: humic acid–vitamin (HV) agar (Hayakawa and Nonomura, 1987a, 1987b), Arthrobacter selective agar (Hagedorn and Holt, 1975), and yeast mannitol agar (YMA) (Vincent, 1970). Regarding the soil suspension to be spread on HV agar, the 10^–2^ diluent containing 0.05% SDS and 6% yeast extract was heated at 40°C for 20‍ ‍min in advance (Hayakawa and Nonomura, 1989). After the heat treatment, bacterial colonies with a similar morphology to the closest relatives of the target ASV sequences were selected. Bacterial colonies cultivated on HV agar, Arthrobacter selective agar, and YMA were re–streaked on ISP agar for isolation (Shirling and Gottlieb, 1966). The isolated bacteria were preserved in 15% glycerol at –‍20°C.

The nearly full–length 16S rRNA sequences of isolated bacteria were elucidated using bacterial cells on ISP medium. In brief, bacterial DNA was extracted from the cell suspension by heating at 99°C for 10‍ ‍min. After centrifugation, the supernatant was treated with PCI followed by CIA, and the aqueous upper layer containing bacterial genomic DNA was used as a template for PCR with the primers 27f (5′–GAGTTTGATCMTGGCTCAG–3′) and 1492r (5′–GGYTACCTTGTTACGACTT–3′) (Ikenaga *et al.*, 2002). PCR conditions were the same as those described above. PCR products were used for cycle sequencing with the internal primer 907r (5′–CCGTCAATTCCTTTGAGTTT–3′) (Ikenaga *et al.*, 2015) to elucidate the upstream sequences of the 16S rRNA genes. DNA sequences were exami­ned using an ABI PRISM 3130xl Genetic Analyzer (Thermo Fisher Scientific). The sequences of bacterial isolates were compared to those of the target ASV, and isolates with identical sequences to the ASV were selected.

The downstream sequences of the 16S rRNA genes were elucidated for the selected isolates using the internal primer 786f (5′–‍GATTAGATACCCTGGTAG–3′) (Wang and Qian, 2009). Based on overlapping sequences upstream and downstream, both sequences were connected to generate the nearly full sequences of‍ ‍16S rRNA genes. Full–length 16S rRNA gene sequences were‍ ‍uploaded and compared with type strains available on the‍ ‍EzBioCloud server (https://www.ezbiocloud.Net/). Multiple alignments were performed with the closest relatives using CLUSTAL W built in MEGA software version 11 (https://www.megasoftware.net). Phylogenetic trees were constructed based on 1,000 resamplings using three different algorithms: neighbor–joining (Saitou and Nei, 1987), maximum parsimony (Fitch, 1971), and maximum likelihood (Felsenstein, 1981). The bacterial 16S rRNA gene sequences obtained from the isolates were registered in Genebank under accession numbers PQ656671 to PQ656702.

### Evaluation of antagonistic activity of selected bacteria against *D. destruens* by diffusible metabolites

The strain of *D. destruens* (MAFF 246953) causing sweet potato foot rot disease was purchased from the National Agriculture and Food Research Organization (NARO). The pathogen was grown on potato dextrose agar (PDA) (4‍ ‍g‍ ‍L^–1^ potato starch, 20‍ ‍g‍ ‍L^–1^ glucose, and 20‍ ‍g‍ ‍L^–1^ agar) at 30°C and the mycelia that grew were preserved in 10% glycerol and 5% trehalose at –20°C. *D. destruens* was inoculated on PDA, sweet potato dextrose (SPDA) (4‍ ‍g‍ ‍L^–1^ sweet potato starch, 20‍ ‍g‍ ‍L^–1^ glucose, and 20‍ ‍g‍ ‍L^–1^ agar), and Koganesangan sweet potato (*Ipomoea batatas*) dextrose agar (KSPDA) to investigate the most effective medium for evaluating antagonistic activity. To prepare KSPDA, small pieces of 200‍ ‍g Koganesangan were boiled, the extract was adjusted to 1 L with distilled water, and 20‍ ‍g‍ ‍L^–1^ glucose and 20‍ ‍g‍ ‍L^–1^ agar were added before sterilization.

To evaluate antagonistic activity, *D. destruens* was initially grown on one of three selected agar media. Pieces of agar with *D. destruens* mycelia that were 7‍ ‍mm in diameter were then placed in the center of fresh agar. After an incubation for 3 days, agar plates with growing mycelia were used for the assay. Isolated bacteria were spread on ISP agar and incubated at 30°C for 3 days. Three pieces of ISP agar that were 7‍ ‍mm in diameter, each with a bacterial colony forming on its surface, were placed 1 to 3‍ ‍cm away from pathogen–containing agar. Plates were prepared in triplicate for each isolate and incubated at 30°C while assessing the growth of *D. destruens* and bacterial antagonistic activity.

### Statistical ana­lysis

A one–way ANOVA followed by Tukey’s HSD post hoc test was performed to test the significance of differences among different organic material treatments at each sampling time. All statistical ana­lyses were performed with R studio version 4.2.3 software (https://cran.r–project.org/bin/windows/base/old/4.2.3/).

## Results and Discussion

### Effects of the application of organic materials on soil bacterial populations

**Fig. 1.**
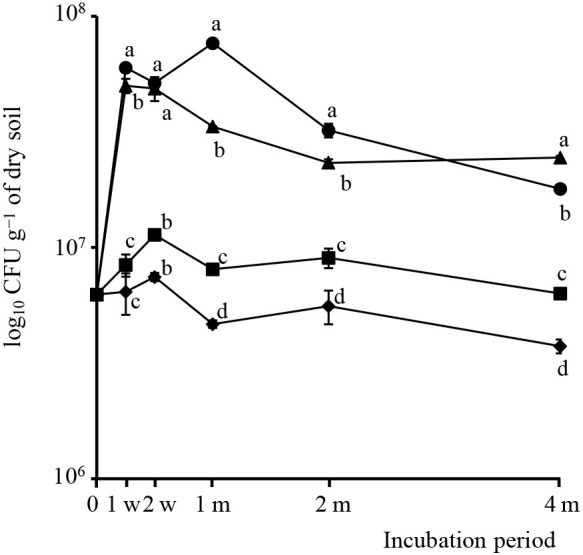
Numbers of soil bacteria in different treatments during the incubation period. These numbers were enumerated by the culture–dependent method. Circles, triangles, squares, and diamonds indicate soils amended with rice bran (RB), Soil–fine (SF), Kuroihitomi (KH), and chemical fertilizer (CF), respectively. Standard deviations were calculated for the numbers of colonies across three replications for each sample. Different letters indicate significant differences at *P*<0.05 within the different treatments at the same sampling time, as assessed by Tukey’s HSD post hoc test.

The responses of bacterial populations in potted soils to the KH, SF, and RB treatments were periodically investigated. The CF treatment was also prepared as a control. The bacterial population, indicated by the total number of bacterial colonies on an agar plate, was larger in soils amended with organic materials (KH, SF, and RB) than in soils treated with CF. As shown in Fig. 1, population dynamics varied over the incubation period based on the treatments applied.

After an incubation for one week, colony numbers significantly increased (approximately 10–fold, *P*<0.05) in SF– and RB–treated soils. Thereafter, colony numbers gradually decreased, but remained higher than in KH– and CF–treated soils incubated for four months. No significant differences were observed in bacterial counts between KH– and CF–treated soils incubated for 1 and 2‍ ‍weeks (*P*>0.05). However, the total number of bacteria in KH–treated soil slightly increased, peaked (2–fold increase) after 2‍ ‍weeks, and was then maintained at a higher level than in CF–treated soil during the incubation periods. No significant differences were observed in the total number of bacteria between CF–treated soil and 0–time soil throughout the incubation period (Supplementary Table S1).

Increases in bacterial populations in amended soils may be attributed to growth promotion by the organic matter applied, which provide accessible nutrients to bacteria in the soil (Bailey and Lazarovits, 2003). The present results indicated that the bacterial populations in SF– and RB–treated soils exhibited stronger responses than those in KH–treated soil within a short period of one week. Fresh organic materials, such as SF and RB, decompose more rapidly than composted organic material, including KH. Easily degradable organic matter undergoes more rapid decomposition during the composting process than in soil (Bell *et al.*, 2003). Therefore, the bacterial populations in KH–treated soil increased slowly, but were still higher than those in CF–treated soil, indicating positive effects on the growth of soil bacteria. The subsequent gradual decrease in bacterial populations throughout the incubation period may have been attributed to the consumption of available organic materials.

### DNA recovery and PCR efficiency with different amounts of casein sodium in DNA extraction from Andosol soil

To increase the efficiency of DNA recovery from soil, the kit protocol for DNA extraction was modified by the addition of casein sodium to prevent DNA from binding to allophane and imogolite in Andosol. Casein, the major protein in milk, is known to improve the yield and quality of DNA extracted from recalcitrant soils (Ikeda *et al.*, 2008). Morimoto *et al.* (2023) also reported better DNA recovery with the addition of casein sodium by comparing skim milk and casein sodium in DNA extraction from allophanic Andosol. In the present study, the optimum concentration was investigated using 0–time soil with different concentrations of casein sodium (0, 5, 10, 15, 20, 25, 30, 35, and 40‍ ‍mg per extraction tube).

**Fig. 2.**
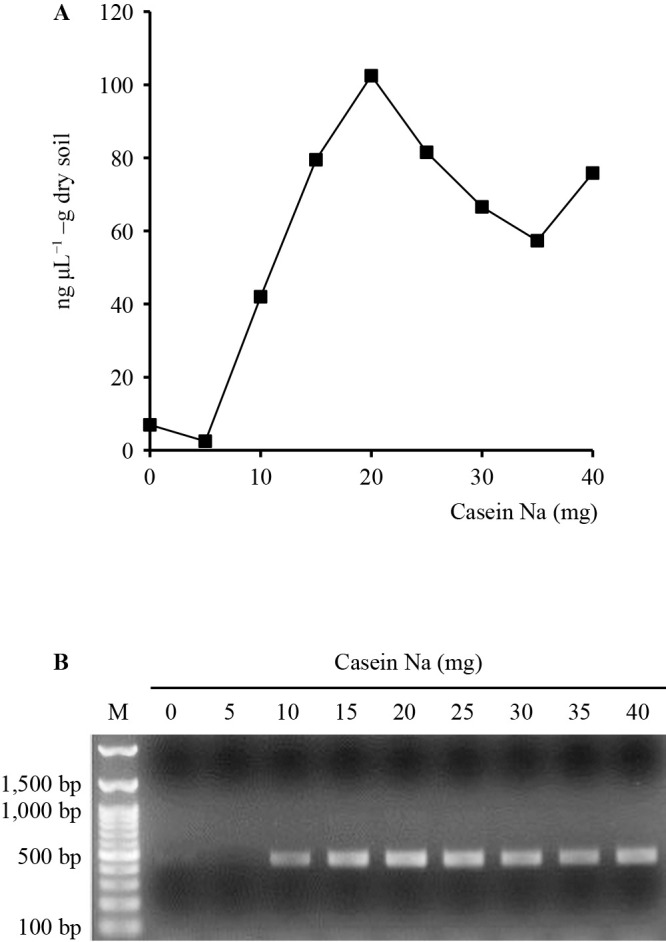
Soil DNA yields and PCR amplification efficacy of bacterial 16S rRNA genes. (**A**) DNA yields from soil extracted with different concentrations of casein Na. (**B**) Gel electrophoresis (1.5% agarose) of PCR products of bacterial 16S rRNA genes amplified with the bacterial primer set 341f and 805r from each extracted soil sample.

As shown in Fig. 2A, DNA was not recovered from soil samples without casein sodium (0‍ ‍mg) or from those with 5‍ ‍mg of casein sodium. However, as the amount of casein sodium increased from 10 to 20‍ ‍mg, DNA yields significantly increased from 7‍ ‍ng μL^–1^ at 0‍ ‍mg up to 103‍ ‍ng μL^–1^ at 20‍ ‍mg, the highest concentration observed. Thereafter, yields gradually decreased. This result was attributed to the reduced recovery of DNA solution after the PCI treatment due to the accumulation of coagulants at the interface during the extraction process.

As shown in Fig. 2B, the intensity of the PCR product of the soil bacterial partial 16S rRNA gene correlated with the result shown in Fig. 2A. The intensities of PCR products were extremely low or below the detection level for soils without casein sodium and with 5‍ ‍mg of casein sodium. The intensities of PCR products were sufficient at concentrations >10‍ ‍mg, with the highest intensity among all tests being observed for 20‍ ‍mg of casein sodium. These results indicate that the addition of 20‍ ‍mg of casein sodium was optimal for successful DNA extraction and PCR amplification in the community ana­lysis of bacteria in Andosol samples.

### Changes in the relative abundance of bacterial communities with the application of organic materials

**Fig. 3.**
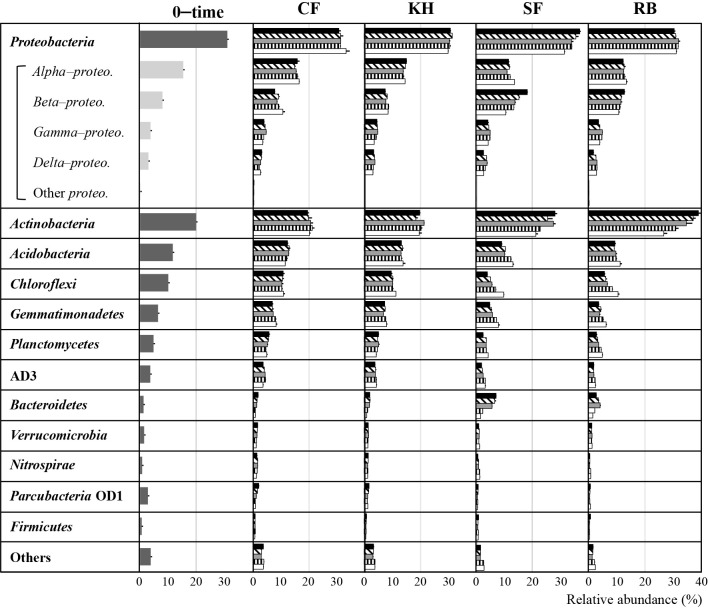
Relative abundance (%) of dominant bacterial phyla in 0–time soil and soils amended with different treatments during incubation periods. The most dominant *Proteobacteria* was additionally indicated at the class level. Standard deviations were calculated for the percentages of relative abundance across three replications for each sample. CF, KH, SF, and RB represent soils amended with chemical fertilizer, Kuroihitomi, Soil–fine, and rice bran, respectively. Symbols in the CF, KH, SF, and RB treatments indicate the following: 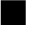 1 week, 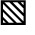 2 weeks, 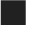 1 month, 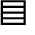 2 months, 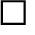 4 months.

Meta 16S rRNA gene sequencing identified 12 bacterial phyla in soil samples, representing >95% of the total (Fig. 3). The 16S rRNA gene, approximately 1,600 base pairs long, has nine hypervariable conservative regions (V1–V9) (Kim *et al.*, 2011; Tringe and Hugenholtz, 2008; Wang and Qian, 2009). Conservative regions help identify higher taxa, while variable regions are useful for distinguishing genera or species. The V3/V4 regions are preferred for their high nucleotide diversity and discriminatory ability.

The other phyla with low abundance (<1% on average) were compiled as “others”. Among the 12 phyla, *Proteobacteria* and *Actinobacteria* were dominant, followed by *Acidobacteria* and *Chloroflexi*. The relative abundance of *Actinobacteria* was approximately 20% in 0–time and KH–treated soils. However, abundance in SF– and RB–treated soils increased to nearly 30 and 40%, respectively, particularly from one week to one month of incubation. The major‍ ‍class of *Proteobacteria* were *Alpha–proteobacteria*, followed by *Beta–proteobacteria*. *Alpha–proteobacteria* accounted for 15.6, 14.7, 13.6, and 13.4% on average of the bacterial populations in CF–, KU–, SF–, RB–treated soils, with no significant differences being observed among treatments throughout the incubation period. Furthermore, no significant differences were observed in the relative abundance of *Beta–proteobacteria* between CF– and KH–treated soils, averaging 9.0 and 8.1%, respectively. However, their relative abundance was higher in SF– and RB–treated soils, accounting for 18.3 and 15.3% in SF–treated soil incubated for one and two weeks, respectively, and 12.7% in RB–treated soil incubated for one week.

The present results showed that the application of SF and RB to soil exerted stronger effects on the soil bacterial community than KH, with marked increases being observed in the relative abundance of *Actinobacteria* and *Beta–proteobacteria*. Bacteria belonging to *Actinobacteria* and *Beta–proteobacteria* are known to be strong decomposers of organic matter (Strap, 2011; Li *et al.*, 2021). Jangid *et al.* (2008) reported that *Actinomycetes*, which belongs to *Actinobacteria*, contributed to decomposition during the early period of an incubation after the application of sewage sludge. *Beta–proteobacteria* were also detected in the earlier stages of litter decomposition (Lima and Colpo, 2013, Zeng *et al.*, 2019). Consistent with previous findings, the most significant responses were observed in the first week of incubation after the application of organic materials. This is presumably due to the SF and RB amendments stimulating the activity of bacteria, such as *Actinobacteria* and *Beta–proteobacteria*, by utilizing the fresh organic materials as an energy source, causing a short–term change known as the “priming effect” (Blagodatskaya and Kuzyakov, 2008). Consistent with previous findings, the quality of fresh organic matter may affect the soil microbial community structure because microbes exhibit substrate preference (Nicolardot *et al.*, 2007). Fresh organic matter with a higher C:N ratio and higher lignin content generally increases the relative abundance of fungi and actinomycetes, which are adapted to nutrient–poor environments (Eskelinen *et al.*, 2009, Gul *et al.*, 2012). The present results indicated that organic fertilization markedly changed the composition of the indigenous soil bacterial community in response to available substrates depending on the amendment materials. In contrast, the impact of inorganic CF alone on the bacterial composition was negligible, with no significant differences from that in 0–time soil.

**Fig. 4.**
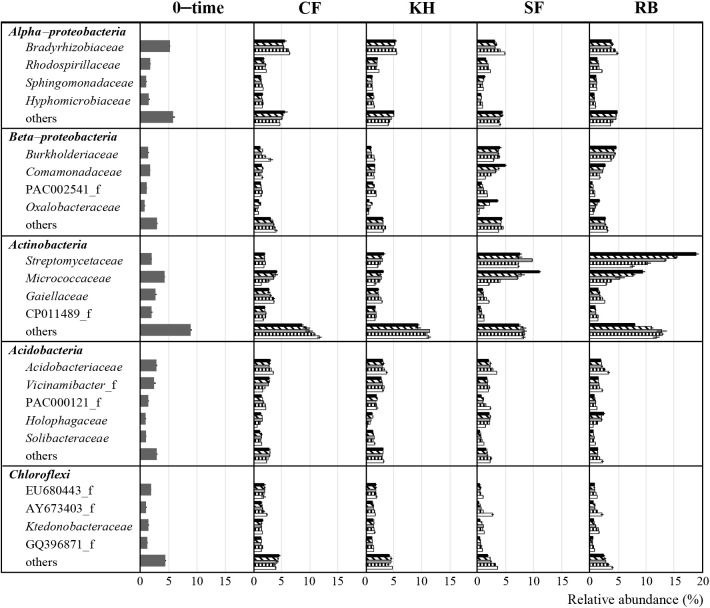
Relative abundance (%) of bacterial families of *Alpha–proteobacteria*, *Beta–proteobacteria*, *Actinobacteria*, *Acidobacteria*, and *Chloroflexi* in 0–time soil and soils amended different treatments during incubation periods. These were the top five dominant bacterial taxa. Standard deviations were calculated for the percentages of relative abundance across three replications for each sample. CF, KH, SF, and RB represent soils amended with chemical fertilizer, Kuroihitomi, Soil–fine, and rice bran, respectively. Symbols in the CF, KH, SF, and RB treatments indicate the following: 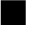 1 week, 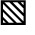 2 weeks, 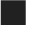 1 month, 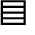 2 months, 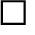 4 months.

To further understand the shift in soil bacterial communities, the major bacterial phyla, *Actinobacteria*, *Alpha–proteobacteria*, *Beta–proteobacteria*, *Acidobacteria*, and *Chloroflexi*, were investigated at the family level. Relative abundance >1% was selected for comparisons. As shown in Fig. 4, *Streptomycetaceae* and *Micrococcaceae*, which belong to *Actinobacteria*, and *Burkholderiaceae*, which belongs to *Beta–proteobacteria*, showed differences in relative abundance between the organic material treatments and 0–time soil. After one week of incubation, *Streptomycetaceae* increased from 2.0% in 0–time soil to 3.2, 7.7, and 18.9% in KH–, SF–, and RB–treated soils, respectively. The relative abundance of *Micrococcaceae* also significantly increased, from 4.3% in 0–time soil to 11.2 and 9.4% in SF– and RB–treated soils, respectively. The relative abundance of *Burkholderiaceae* increased from 2.0% in 0–time soil to 4.1 and 4.8% in SF– and RB–treated soils, respectively. Therefore, relative abundance increased in the first week, and then generally decreased during the incubation. The family level abundance of *Acidobacteria* and *Chloroflexi* remained unchanged with the different treatments and over time.

The present results indicate that SF and RB increased soil nutrient contents as rapidly available substrates more than KH and promoted the growth of *Streptomycetaceae*, *Micrococcaceae*, and *Burkholderiaceae*, which may have the ability to decompose readily available soil organic matter (Fontaine *et al.*, 2003; Pérez-Piqueres *et al.*, 2006; Sinsabaugh *et al.*, 2008). Consistent with previous findings, these activated bacterial families have been shown to produce extracellular enzymes that decompose easily assimilable compounds of straw (Chen *et al.*, 2021). This may be explained by lower nutrient concentrations in KH than in RB and SF due to the poorer decomposability of organic material in compost. Available organic material may have already been decomposed during composting processes, resulting in recalcitrant compounds remaining (Wang *et al.*, 2004). In addition, *Streptomycetaceae* and *Burkholderiaceae* were not dominant in the organic materials of KH, SF, and RB themselves (Supplementary Table S2). Although the relative abundance of *Micrococcaceae* was 10.5% in KH and 0.7% in SF, they differed at the ASV sequence level.

### Changes in the relative abundance of *Streptomycetaceae*, *Micrococcaceae*, and *Burkholderiaceae* at the ASV level

Among *Streptomycetaceae*, *Micrococcaceae*, and *Burkholderiaceae*, the differentiation of bacterial communities was further investigated at the ASV level. As shown in Table 1, ASV002 and ASV005 in *Streptomycetaceae*, ASV001 in *Micrococcaceae*, and ASV007 in *Burkholderiaceae* were the dominant ASVs. Closely related bacteria based on the ASV sequences derived from the V3/V4 region of the 16S rRNA gene, elucidated by next–generation sequencing, belonged to the genera of *Kitasatospora*–*Streptomyces* for ASV002 and ASV005, *Arthrobacter*–*Pseudarthrobacter* for ASV001, and *Paraburkholderia* for ASV007. During the incubation, the relative abundance of these ASVs was increased by the organic amendments.

**Fig. 5.**
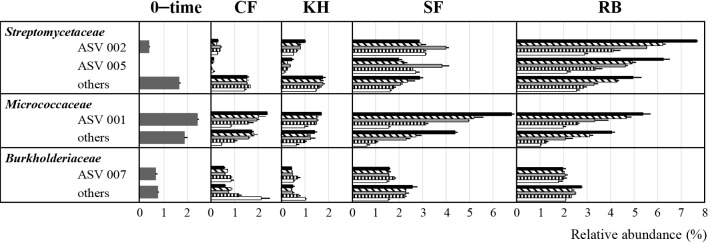
Relative abundance (%) of ASVs in *Streptomycetaceae*, *Micrococcaceae*, and *Burkholderiaceae* in 0–time soil and soils amended with different treatments during incubation periods. The relative abundance of three bacterial families increased with the addition of organic materials. Standard deviations were calculated for the percentages of relative abundance across three replications for each sample. CF, KH, SF, and RB represent soils amended with chemical fertilizer, Kuroihitomi, Soil–fine, and rice bran, respectively. Symbols in the CF, KH, SF, and RB treatments indicate the following: 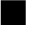 1 week, 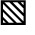 2 weeks, 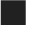 1 month, 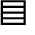 2 months, 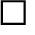 4 months.

A focus on each ASV (Fig. 5) revealed that the relative abundance of ASV002 in CF–treated soil did not markedly differ from that in 0–time soil throughout the incubation period. On the other hand, its relative abundance on average, which was 0.4% in 0–time soil, increased to 1.0, 2.9, and 7.7% in KH–, SF–, and RB–treated soils, respectively, after one week of cultivation. Although its relative abundance gradually decreased with these treatments, SF– and RB–treated soils continued to show a high abundance of approximately 3% even after four months. Regarding ASV005, it showed a smaller relative abundance than ASV002, but exhibited similar dynamics. ASV001 showed a smaller difference in relative abundance among 0–time, CF–, and KH–treated soils, remaining at approximately 2%. In contrast, in SF– and RB–treated soils, significant increases to 6.8 and 5.4%, respectively, were observed after one week of cultivation. ASV007 showed similar changes to ASV001, with relative abundance <1% in 0–time, CF–, and KH–treated soils. However, increases were observed in SF– (1.5%) and RB–treated soils (2.0%) that persisted throughout the incubation period. As described above, each ASV responded differently depending on the treatments, with ASV002 and ASV005 having higher relative abundance in RB–treated soil than in SF–treated soil, whereas the relative abundance of ASV001 was higher in SF–treated soil than in RB–treated soil.

### Isolation of bacteria with the same sequence as ASVs whose relative abundance increased with the application of organic materials

Based on bacterial phylogenetic positions, selective agar media were used to isolate bacteria, targeting *Kitasatospora*–*Streptomyces*, *Arthrobacter*–*Pseudarthrobacter*, and *Paraburkholderia*. Bacteria with identical sequences to these ASVs were selected by sequencing the upstream region of 16S rRNA genes containing the V3 and V4 regions. The full–length 16S rRNA genes of the relevant isolates were then elucidated. As shown in Table 1, among 122 isolates, 11 had identical sequences with ASV002, 10 with ASV005, 8 with ASV001, and 3 with ASV007. These isolates were further divided into 7, 6, 4, and 1 group for ASV002, ASV005, ASV001 and ASV007, respectively, based on the full–length sequences of the 16S rRNA genes (Table 2).

**Fig. 6.**
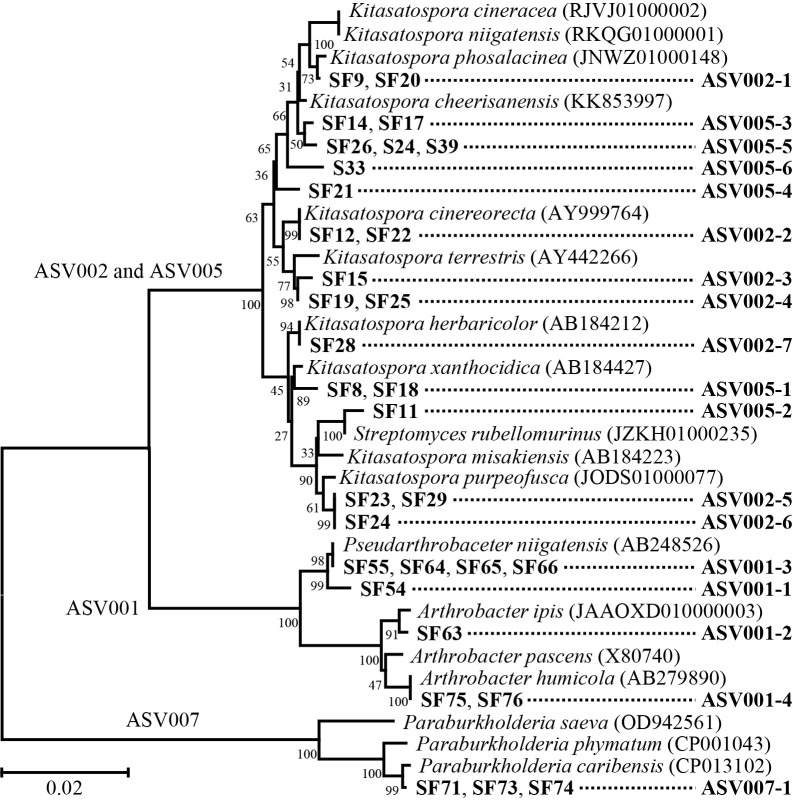
Neighbor–joining phylogenetic tree based on 16S rRNA gene sequences of isolates in respective ASV groups, which have the same sequences as ASV002, ASV005, ASV001, and ASV007 in V3–V4 regions identified by MiSeq high throughput sequencing. The number of nodes indicate bootstrap values calculated based on a dataset resampled 1,000 times. The bar represents 0.01 substitutions per nucleotide position. Sequences of at least 1,400 nt were used for the calculation.

The ASV002 and ASV005 groups were closely affiliated with the genus *Kitasatospora*, while only ASV005–2 was affiliated with the genus *Streptomyces*. A phylogenetic ana­lysis of 16S rRNA gene sequences previously showed that members of the genus *Kitasatospora* form a tight cluster with *Streptomyces*, but represent a distinct and legitimate genus separate from *Streptomyces* (Girard *et al.*, 2014; Takahashi, 2017). As shown in Fig. 6, the isolate positions of the respective groups with the same sequences as ASV002 and ASV005 formed the same cluster with each other; however, clear positional relationships were not observed between the sequences of ASV002 and ASV005 because even if the ASV sequences are the same, phylogenetic variations from full–length 16S rRNA gene sequences occur. Although isolates were grouped based on their sequences, some of the groups were phylogenetically close to each other, and their closest relatives were also the same.

The closest relatives in the ASV002 groups were *Kitasatospora phosalacinea* NRRL B–16230^T^ (JNWZ01000148) for ASV002–1, *Kitasatospora cinereorecta* JCM 6916^T^ (AY999764) for ASV002–2, *Kitasatospora terrestris* HKI 0186^T^ (AY442266) for ASV002–3 and ASV002–4, *Kitasatospora misakiensis* NBRC 12891^T^ (AB184223) for ASV002–5, *Kitasatospora purpeofusca* NRRL B–1817^T^ (AB184427) for ASV002–6, and *Kitasatospora herbaricolor* NBRC 12876^T^ (AB184212) for ASV002–7. The closest relatives in the ASV005 groups were *Kitasatospora xanthocidica* NBRC 13469^T^ (AB184427) for ASV005–1, *Streptomyces rubellomurinus* ATCC 31215^T^ (JZKH01000235) for ASV005–2, and *Kitasatospora cheerisanensis* KCTC 2395^T^ for ASV005–3, ASV005–4, ASV005–5, and ASV005–6. *Kitasatospora* are regarded as biological control agents due to their potential to produce a wide range of secondary substances. At least 50 bioactive compounds have so far been identified from strains of *Kitasatospora*, such as propioxatins (enkephalinase B inhibitor), terpentecin (antitumor activity) (Takahashi, 2017), setamicin (nematocidal and antifungal activities), and fosalacin (herbicide activity) (Yun *et al.*, 2020), as well as endophenaside antibiotics (Wu *et al.*, 2015). *Kitasatospora* species were previously shown to exhibit antagonistic activity against plant pathogens, such as *Phytophthora citricola*, *Rhizoctonia solani*, and rice seedling diseases (Haesler *et al.*, 2008; Yun *et al.*, 2020; Tu *et al.*, 2023). *Streptomyces* are also regarded as biological control agents, with secondary substances including vitamins, alkaloids, plant growth factors, enzymes, and enzyme inhibitors (Rothrock and Gottlieb, 1984; Lehr *et al.*, 2008). For example, an RB treatment of soil was shown to increase the population of non–pathogenic *Streptomyces* spp., eventually decreasing pathogenic *Streptomyces* spp. caused by potato common scab (Tomihama *et al.*, 2016). Another study reported excellent biological control activity against phytopathogenic soil–borne fungi, such as *R. solani* and *Phytophthora* spp. (Rothrock and Gottlieb, 1981). Furthermore, volatile organic compounds (VOCs) emitted by some *Streptomycetes* spp. exhibited strong antifungal activity and significantly inhibited the growth of *Ceratocyctis fimbriata*, which causes sweet potato black rot (Li *et al.*, 2020; Gong *et al.*, 2022; Zhang *et al.*, 2024).

The closest relatives of ASV001 were divided into two bacterial genera: *Pseudarthrobacter* and *Arthrobacter*. The closest relative of both ASV001–1 and ASV001–3 was *Pseudarthrobacter niigatensis* LC4^T^ (AB248526), and those of ASV001–2 and ASV001–4 were *Arthrobacter ipis* IA7^T^ (JAAOXD010000003) and *Arthrobacter humicola* KV–653^T^ (AB279890), respectively. The isolates in ASV007 showed the same sequences for their full–length 16S rRNA genes, and their closest relative was *Paraburkholderia caribensis* MWAP64^T^ (CP013102). *Arthrobacter* strains possess several growth–promoting traits, including high indole–3–acetic acid (IAA) and siderophore production as well as nitrogen fixation. They also produce antibacterial compounds (*e.g.*, dimethylhexadecylamine and arthroamide) against plant pathogens, such as *Xanthomonas campestris* pv. *campestris* and *Fusarium proliferatum*, which cause rice spikelet rot (Velázquez-Becerra *et al.*, 2013; Igarashi *et al.*, 2015; Chhetri *et al.*, 2022). Bacteria in the *Pseudarthrobacter* genus are characterized by nitrogen fixation, phosphate solubilization, metabolic versatility, and plant growth–promoting activity (Suárez-Moreno *et al.*, 2012; Kaur *et al.*, 2016; Hsu *et al.*, 2018). Additionally, previous studies demonstrated that *Paraburkholderia* enhanced plant growth, yield, and antioxidant levels and induced defenses against various pathogens, including *X. campestris*, *Botrytis cinerea*, and *Fusarium solani* (Rahman *et al.*, 2018; Jeon *et al.*, 2021; Gu *et al.*, 2023). Therefore, these dominant bacteria appear to exhibit antifungal activities against *D. destruens*, which causes sweet potato foot rot disease.

### Antagonistic activity of isolates against *D. destruens* by the confrontation culture method

**Fig. 7.**
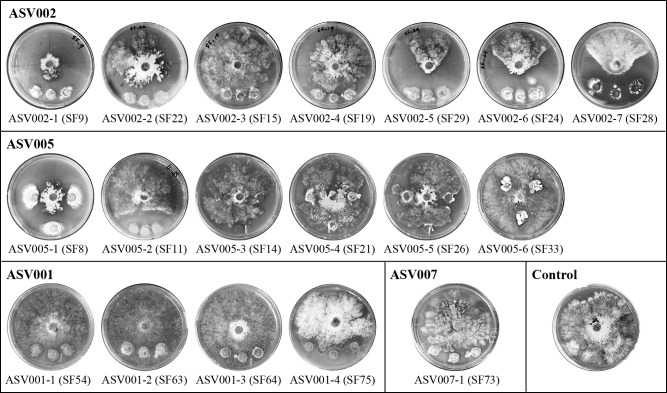
Representative photographs of confrontation cultures in which isolates from respective groups and *Diaporthe destruens* were co–cultivated during their development. The mycelia of *D. destruens* were placed in the center of agar plates, and colonies of the isolates were placed 1 to 3‍ ‍cm away from the pathogen.

Limited research has been conducted on microbial antagonistic activity against *D. destruens*. Tsurumaru *et al.* (Tsurumaru, H., *et al.* 2023 Characterization of *Diaporthe destruens*, the pathogen causing sweet potato foot rot disease, and exploration of growth–inhibiting bacteria. Jpn Soci Soil Microbiol 2023, Matsudo Chiba Japan, Jun 10–11 2023) screened bacteria from soil samples and isolated antagonistic bacteria against *D. destruens*. However, since these bacteria were not from indigenous environments, it remains unclear whether they will be incorporated into the soil bacterial network and exhibit the expected antagonistic function when inoculated into soil. To overcome this issue, it is considered effective to promote their growth using organic material amendments without affecting the original soil bacterial network. We herein investigated whether the isolates obtained exhibited the potential for antagonistic activity based on the above–described hypothesis. Fig. 7 shows representative photographs of the confrontation culture method, in which isolates from the respective groups and *D. destruens* were both grown together during their development. Other photographs are shown in Supplementary Fig. S1.

Among the isolate groups of ASV002 and ASV005, ASV002–5 (SF23 and SF29), ASV002–6 (SF24), and ASV002–7 (SF28) showed visually noticeable inhibitory zones against *D. destruens*. In the groups ASV002–1 (SF9 and SF20), ASV002–2 (SF12 and SF22), and ASV005–1 (SF8 and SF18), the mycelial development of *D. destruens* was inhibited. ASV005–2 (SF11) exhibited weaker antagonistic activity. In the groups ASV001 and ASV007, ASV001–4 (SF75 and SF76) also exhibited weak antagonistic ability. However, an inhibition zone against *D. destruens* was not observed in the isolate groups ASV001–1 (SF54), ASV001–2 (SF63), ASV001–3 (SF55, SF64, SF65, and SF66), and ASV007–1 (SF71, SF73, and SF74). These results clearly suggest that the application of organic materials to soil indirectly promoted the relative abundance of indigenous soil bacteria, particularly *Kitasatospora* spp., which produced bioactive compounds against *D. destruens*.

According to Sosio *et al.* (2000), each actinomycete genetically has the potential to produce between approximately 10 and 20 different secondary metabolites. These metabolites include a wide range of chemical properties and may be utilized for the biological control of pathogens in agriculture. *K. misakiensis*, the closest relative of ASV002–5 (SF23 and SF29), is known for its strong antibacterial and antifungal properties. It produces a number of secondary metabolites that inhibit soilborne fungal diseases caused by *Aspergillus fumigatus* and *Aspergillus niger* (Abdelaziz *et al.*, 2023). Similarly, *K. purpeofusca*, the closest relative of ASV002–6 (SF24), exhibited strong inhibitory activity against a number of pathogens, such as *Escherichia coli*, *Staphylococcus aureus*, *Mycobacterium tuberculosis*, and *Bacillus subtilis* (Zhang *et al.*, 2023). When co–infested with the antagonist and pathogen in soil, *K. herbaricolor*, the closest relative of ASV002–7 (SF28), reduced plant diseases caused by *R. solani* and *Phytophthora megasperma* var. *soja* (Rothrock and Gottlieb, 1981). Therefore, the closest relatives of the isolates that exhibit a large growth inhibitory capacity have been shown to suppress the growth of plant pathogenic fungi, but not the growth of *D. destruens*. Other isolate groups, such as *K. xanthocidica*, the closest relative of ASV005–1 (SF8 and SF18), *S. rubellomurinus*, the closest relative of ASV005–2 (SF11), and *A. humicola*, the closest relative of ASV001–4 (SF75 and SF76), also inhibited the growth of plant pathogens through secondary metabolites and other functions (Okuhara *et al.*, 1980; Kang *et al.*, 2016; Gomez *et al.*, 2023; Jeon *et al.*, 2023). These findings indicate that the application of organic materials increased the relative abundance of indigenous beneficial bacteria, which inhibited the growth of *D. destruens*, particularly with the fresh organic matter SF and RB rather than with composted KH. Previous studies demonstrated that the addition of readily available carbon in the form of organic amendments (fresh or dried plant material) to soil stimulates overall microbial activity (Stark *et al.*, 2008; Michel and Lazzeri, 2011). However, the application of fresh organic matter may sometimes temporarily increase some soil–borne diseases (Hoitink and Boehm, 1999) and lead to competition for oxygen due to heightened macro– and microbial activities. Therefore, planting or sowing a new crop immediately after the application of fresh organic matter needs to be avoided. Fresh organic material is quickly mineralized, and various forms of mineral nitrogen may affect soil pathogens (Huber and Watson, 1974). Nutrients from fresh organic matter may indirectly enhance plant health and yield in subsequent crops. Furthermore, bacterial isolates in some ASV groups did not exhibit growth inhibitory activity. Irrespective of this activity, bacteria exhibiting increases in relative abundance following the addition of organic materials must have a high affinity for these materials. Therefore, the combination of organic materials and antagonistic bacteria prepared as microbial materials will be a more promising method for suppressing the growth of *D. destruens* in the sweet potato field, while utilizing the indigenous soil microbial network from the perspective of sustainable agriculture.

In conclusion, our final goal is to regulate the growth of *D. destruens* in sweet potato fields from a microbiological perspective. This study was part of our comprehensive research. The routes of infection for this disease include field soil and seedlings; the present study focused on soil infection and conducted experiments accordingly. The results obtained herein demonstrated that the addition of organic materials to soil increased the relative abundance of specific bacteria that inhibit the growth of *D. destruens*. This increase was significant with the SF and RB treatments. Although the increase observed with the KH treatment was smaller, the number of bacteria enumerated by cultivated colonies was approximately two–fold higher than that with the CF treatment as a control. Therefore, the actual increase in soil may be estimated by integrating both the bacterial number and their relative abundance, suggesting that the increase is more than that observed by individual ana­lyses. Since KH is a compost, in contrast to the fresh organic materials SF and RB, it may be easier to use in the field than SF and RB.

The present results also revealed that the relative abundance of bacterial isolates with ASV sequences that inhibited the growth of *D. destruens* increased in KH–, SF–, and RB–treated soils, although the ratio of this increase varied. In addition, their relative abundance remained elevated in soils incubated for four months. Although it remains unclear whether the application of organic materials is directly effective for regulating *D. destruens* in sweet potato fields, depending on how the bacterial isolates are utilized, the desirable results, namely, suppressing the development of this disease, which is observed in the late growth stage of sweet potato, may be achieved. Our research group is currently planning to apply antagonistic bacteria as a microbial material in combination with organic materials to sweet potato cultivating fields. The results obtained in the present study provide novel prospectives for solving the spread of disease by *D. destruens*.

## Citation

Soe, Z. M., Sakai, M., Kihara, S., Fukahori, D., Nakamura, M., Ueno, D., et al. (2025) Responses of Soil Bacteria Communities to Organic Material Application and Their Antagonistic Activity against *Diaporthe destruens* Causing Sweet Potato Foot Rot Disease. *Microbes Environ ***40**: ME25011.

https://doi.org/10.1264/jsme2.ME25011

## Supplementary Material

Supplementary Material

## Figures and Tables

**Table 1.** Phylogenetic affiliation of ASV sequences, identified by EzBioCloud (https://www.ezbiocloud.net).

ASV number	Closest bacterial family	Closest bacterial genera	Similarity	Alignment
ASV002	*Streptomycetaceae*	*Kitasatospora* spp., *Streptomyces* spp.	100%	407/407
ASV005	*Streptomycetaceae*	*Kitasatospora* spp., *Streptomyces* spp.	100%	407/407
ASV001	*Micrococcaceae*	*Arthrobacter* spp., *Pseudarthrobacter* spp.	100%	407/407
ASV007	*Burkholderiaceae*	*Paraburkholderia* spp.	100%	427/427

**Table 2.** Closest relatives of bacterial isolates with 16S rRNA gene sequences identical to ASV002, ASV005, ASV001, and ASV007 in relevant regions.

ASV groups	Closest Relatives	Accession Number	Similarity (%)	Sequence (bp)	Alignments	Names of Isolates
ASV002-1	*Kitasatospora phosalacinea* NRRL B-16230^T^	JNWZ01000148	99.86	1,439	1,437/1,439	SF9, SF20
ASV002-2	*Kitasatospora cinereorecta* JCM 6916^T^	AY999764	100	1,439	1,409/1,409	SF12, SF22
ASV002-3	*Kitasatospora terrestris* HKI 0186^T^	AY442266	99	1,441	1,393/1,407	SF15
ASV002-4	*Kitasatospora terrestris* HKI 0186^T^	AY442266	99.22	1,437	1,396/1,407	SF19, SF25
ASV002-5	*Kitasatospora misakiensis* NBRC 12891^T^	AB184223	99.45	1,446	1,436/1,444	SF23, SF29
ASV002-6	*Kitasatospora purpeofusca* NRRL B-1817^T^	AB184427	99.3	1,444	1,435/1,444	SF24
ASV002-7	*Kitasatospora herbaricolor* NBRC 12876^T^	AB184212	100	1,448	1,445/1,445	SF28
ASV005-1	*Kitasatospora xanthocidica* NBRC 13469^T^	AB184427	99.58	1,444	1,422/1,428	SF8, SF18
ASV005-2	*Streptomyces rubellomurinus* ATCC 31215^T^	JZKH01000235	99.58	1,446	1,439/1,445	SF11
ASV005-3	*Kitasatospora cheerisanensis* KCTC 2395^T^	KK853997	99.65	1,437	1,432/1,437	SF14, SF17
ASV005-4	*Kitasatospora cheerisanensis* KCTC 2395^T^	KK853997	99.09	1,436	1,423/1,436	SF21
ASV005-5	*Kitasatospora cheerisanensis* KCTC 2395^T^	KK853997	99.58	1,439	1,433/1,439	SF26, S24, S39
ASV005-6	*Kitasatospora cheerisanensis* KCTC 2395^T^	KK853997	98.94	1,415	1,400/1,415	S33
ASV001-1	*Pseudarthrobacter niigatensis* LC4^T^	AB248526	99.31	1,444	1,434/1,444	SF54
ASV001-2	*Arthrobacter ipis* IA7^T^	JAAOXD010000003	99.65	1,447	1,441/1,446	SF63
ASV001-3	*Pseudarthrobacter niigatensis* LC4^T^	AB248526	99.86	1,444	1,442/1,444	SF55, SF64, SF65, SF66
ASV001-4	*Arthrobacter humicola* KV-653^T^	AB279890	100	1,449	1,442/1,442	SF75, SF76
ASV007-1	*Paraburkholderia caribensis* MWAP64^T^	CP013102	99.38	1,453	1,444/1,453	SF71, SF73, SF74

Each ASV sequence was further divided into 7, 6, 4, and 1 groups for ASV002, ASV005, ASV001, and ASV007, respectively, based on the almost full–length sequences of bacterial 16S rRNA genes.
