# Supplementary material for: Responses of Soil Bacteria Communities to Organic Material Application and Their Antagonistic Activity against Diaporthe destruens Causing Sweet Potato Foot Rot Disease
Source: Microbes Environ. 2025 Sep 6;40(3):ME25011. doi: 10.1264/jsme2.ME25011 (PMC12501867; doi:10.1264/jsme2.ME25011)
Supplement: Supplementary file 1 — Supplementary Material [file 40_25011_s1.pdf]

Supplementary Table S1 Numerical data of Fig. 1 showing the numbers of soil bacteria in different treatments during the incubation period. Those numbers were enumerated by culture–dependent method

Timeline	CH treatmnt	KH treamtmet	SF treamtment	RB treatment
0-time	$6.26 \times 10^6 \pm 3.13 \times 10^5$ ab	$6.26 \times 10^6 \pm 3.13 \times 10^5$ c	$6.26 \times 10^6 \pm 3.13 \times 10^5$ d	$6.26 \times 10^6 \pm 3.13 \times 10^5$ f
1w	$6.43 \times 10^6 \pm 1.60 \times 10^6$ ab	$8.40 \times 10^6 \pm 1.13 \times 10^6$ b	$5.01 \times 10^7 \pm 4.39 \times 10^6$ a	$5.99 \times 10^7 \pm 2.89 \times 10^6$ b
2w	$7.46 \times 10^6 \pm 3.72 \times 10^5$ ab	$1.13 \times 10^7 \pm 3.75 \times 10^5$ a	$4.87 \times 10^7 \pm 7.00 \times 10^6$ a	$5.15 \times 10^7 \pm 3.66 \times 10^6$ c
1m	$4.66 \times 10^6 \pm 2.12 \times 10^5$ cd	$8.05 \times 10^6 \pm 4.29 \times 10^5$ b	$3.33 \times 10^7 \pm 1.37 \times 10^5$ b	$7.65 \times 10^7 \pm 1.94 \times 10^6$ a
2m	$5.58 \times 10^6 \pm 1.14 \times 10^6$ bc	$9.02 \times 10^6 \pm 1.07 \times 10^6$ b	$2.32 \times 10^7 \pm 1.03 \times 10^6$ c	$3.21 \times 10^7 \pm 2.67 \times 10^6$ d
4m	$3.74 \times 10^6 \pm 3.16 \times 10^5$ d	$6.32 \times 10^6 \pm 4.23 \times 10^5$ c	$2.45 \times 10^7 \pm 4.61 \times 10^5$ c	$1.79 \times 10^7 \pm 6.07 \times 10^5$ e
P value	2.0×10^{-3}	1.4×10^{-5}	1.56×10^{-8}	8.12×10^{-13}
CV %	14.79	8.62	10.98	5.77

Standard deviations were calculated for the numbers of colonies across three replications for each sample. Different letters indicate significant differences at a $p < 0.05$ level within the different treatments at the same sampling time, as determined by Tukey's HSD post hoc test. The coefficient variation (the ratio of the standard deviation to the mean) (CV) value describes the variability of the number of soil bacteria in treatments during incubation period.

Supplementary Table S2 The relative abundances (%) of respective bacterial ASVs in soil of 0–time, soils amended with CF, KH, SF, and RB, and organic materials of KH, SF, and RB in the families of Micrococcaceae, Streptomycetaceae, and Burkholderiaceae.

Family	ASV	0–time soil	CF treatment					KH treatment						SF treatment						RB treatment					
			1w	2w	1m	2m	4m	KH itself	1w	2w	1m	2m	4m	SF itself	1w	2w	1m	2m	4m	RB itself	1w	2w	1m	2m	4m
	ASV_011	0.67±0.08	0.62±0.01	0.65±0.09	0.68±0.07	0.49±0.04	0.23±0.02	0±0	0.59±0.03	0.43±0.02	0.51±0.05	0.46±0.10	0.35±0.03	0±0	1.41±0.15	0.93±0.12	0.85±0.08	0.42±0.03	0.31±0.05	0±0	1.61±0.09	1.20±0.03	0.81±0.15	0.58±0.09	0.44±0.03
	ASV_104	0.18±0.03	0.15±0.03	0.18±0.04	0.15±0.04	0.03±0.04	0±0	0±0	0.14±0.01	0.10±0.07	0.09±0.07	0.10±0.07	0±0	0±0	0.55±0.02	0.31±0.03	0.25±0	0.17±0.05	0.08±0.06	0±0	0.38±0.03	0.26±0.05	0.20±0.04	0.15±0.01	0.14±0.04
	ASV_020	0±0	0±0	0±0	0±0	0±0	0±0	10.54±0.03	0±0	0±0	0±0	0±0	0±0	0.66±0.17	0±0	0±0	0±0	0±0	0±0	0±0	0±0	0±0	0±0	0±0	0±0
	ASV_001	2.44±0.06	2.39±0.02	2.02±0.30	1.95±0.11	1.59±0.23	0.85±0.05	0±0	1.70±0.03	1.49±0.09	1.52±0.06	1.48±0.04	1±0.15	0±0	6.78±0.12	5.18±0.4	4.94±0.04	3.13±0.11	1.55±0.07	0±0	5.38±0.31	4.66±0.22	3.32±0.58	2.6±0.07	1.98±0.12
Micrococcaceae	ASV_030	0.43±0.01	0.40±0.05	0.43±0.05	0.34±0.01	0.25±0.01	0.23±0.06	0±0	0.26±0.02	0.28±0.05	0.26±0.04	0.22±0.02	0.16±0.02	0±0	0.78±0.06	0.59±0.04	0.50±0.05	0.24±0.03	0.09±0.07	0±0	0.67±0.04	0.51±0.04	0.34±0.07	0.14±0.10	0.14±0.02
	ASV_045	0.29±0.03	0.26±0.03	0.27±0.02	0.20±0.01	0.16±0.04	0±0	0±0	0.20±0.04	0.18±0.03	0.17±0.01	0.15±0.03	0.09±0.07	0±0	0.76±0.03	0.48±0.05	0.42±0.09	0.15±0.01	0.13±0.10	0±0	0.56±0.03	0.48±0.01	0.33±0.03	0.20±0.03	0.14±0
	ASV_096	0.21±0.05	0.19±0.04	0.20±0.05	0.16±0.01	0.05±0.07	0±0	0±0	0.17±0.05	0.10±0.08	0.15±0.04	0.09±0.06	0.04±0.06	0±0	0.54±0.03	0.27±0.04	0.20±0.02	0±0	0±0	0±0	0.45±0.02	0.37±0.02	0.24±0.01	0.18±0.11	0.16±0.01
	ASV_328	0.11±0.05	0.11±0.03	0.11±0	0.06±0.04	0.02±0.03	0±0	0±0	0.05±0.04	0.02±0.02	0.04±0.03	0±0	0±0	0±0	0.14±0.02	0.11±0.02	0.04±0.03	0.02±0.03	0±0	0±0	0.23±0.03	0.19±0.03	0.11±0.01	0.02±0.03	0±0
	Other ASVs	0±0	0±0	0±0	0±0	0±0	0±0	0±0	0±0	0±0	0.02±0.02	0±0	0±0	0.08±0.03	0.20±0.05	0.03±0.04	0.03±0.03	0.01±0.02	0±0	0±0	0.16±0.04	0.10±0.08	0.03±0.03	0±0	0±0
	ASV_002	0.38±0.04	0.30±0.03	0.25±0.03	0.41±0.06	0.38±0.04	0.28±0.04	0±0	1.02±0.04	0.77±0.08	0.82±0.02	0.67±0.05	0.52±0.02	0±0	2.85±0.01	2.92±0.22	3.98±0.13	3.04±0.11	3.11±0.06	0±0	7.65±0.05	6.20±0.13	5.52±0.03	4.11±0.30	2.90±0.14
	ASV_005	0±0	0.12±0.01	0.09±0.07	0±0	0.03±0.04	0.1±0.08	0±0	0.46±0.09	0.26±0.02	0.38±0.04	0.27±0.02	0.12±0.09	0±0	1.97±0.15	2.19±0.15	3.82±0.31	2.55±0.04	2.69±0.17	0±0	6.24±0.26	4.91±0.17	4.62±0.10	3.35±0.29	2.15±0.17
	ASV_014	0.49±0.02	0.45±0.02	0.47±0.03	0.47±0.01	0.46±0.01	0.46±0.01	0±0	0.37±0.02	0.39±0.05	0.4±0.03	0.40±0.03	0.41±0.01	0±0	0.55±0.11	0.46±0.07	0.46±0.08	0.44±0.07	0.34±0.01	0±0	1.30±0.07	1.17±0.15	0.81±0.01	0.84±0.08	0.89±0.03
	ASV_070	0.29±0.06	0.29±0.08	0.28±0.02	0.29±0.05	0.29±0.05	0.30±0.10	0±0	0.24±0.03	0.24±0.07	0.23±0.07	0.32±0.06	0.27±0.09	0±0	0.14±0.10	0.19±0.03	0.21±0.04	0.18±0.02	0.24±0.03	0±0	0.15±0.06	0.08±0.11	0.17±0.13	0.15±0.08	0.09±0.13
	ASV_089	0.11±0.08	0.22±0.04	0.20±0.01	0.14±0.04	0.15±0.11	0.12±0.08	0±0	0.17±0.03	0.17±0.04	0.18±0.02	0.22±0.01	0.19±0	0±0	0.23±0	0.10±0.08	0.18±0.01	0.23±0.04	0.14±0.06	0±0	0.40±0.02	0.24±0.03	0.20±0.04	0.17±0.03	0.19±0.10
	ASV_111	0.18±0.02	0.18±0.02	0.21±0.03	0.18±0.02	0.24±0.04	0.22±0.01	0±0	0.17±0.03	0.15±0.01	0.08±0.06	0.11±0.08	0.21±0.03	0±0	0.10±0.15	0.22±0.02	0.20±0.04	0.11±0.09	0.05±0.07	0±0	0.21±0.03	0.2±0.04	0.17±0.03	0.15±0.03	0.14±0.04
	ASV_160	0±0	0±0	0±0	0±0	0.03±0.04	0±0	0±0	0±0	0±0	0±0	0.02±0.03	0±0	0±0	0.29±0.05	0.27±0.08	0.24±0.02	0.14±0.10	0.21±0.03	0±0	0.28±0.03	0.29±0.04	0.29±0.03	0.31±0.08	0.16±0.01
Streptomycetaceae	ASV_161	0±0	0±0	0±0	0±0	0±0	0.03±0.04	0±0	0.09±0.02	0.03±0.05	0±0	0±0	0±0	0±0	0.12±0.09	0.11±0.08	0.04±0.06	0.08±0.06	0.13±0.01	0±0	0.47±0.10	0.45±0.01	0.35±0.08	0.28±0.03	0.25±0.03
	ASV_196	0.12±0.02	0.11±0.03	0.07±0.05	0.10±0.01	0.04±0.06	0.09±0.07	0±0	0.10±0.01	0.09±0.04	0.08±0.01	0.11±0.01	0.03±0.04	0±0	0.23±0.07	0.09±0.07	0.07±0.05	0.06±0.04	0.09±0.04	0±0	0.25±0.05	0.17±0.02	0.12±0.01	0.06±0.04	0.11±0.03
	ASV_216	0±0	0±0	0±0	0.01±0.01	0±0	0±0	0±0	0±0	0±0	0±0	0±0	0±0	0±0	0.16±0.01	0.13±0.01	0.15±0.02	0.14±0.03	0.16±0.04	0±0	0.3±0.01	0.29±0.02	0.22±0.04	0.24±0.02	0.19±0.03
	ASV_229	0.09±0.07	0.07±0.05	0.06±0.05	0.05±0.04	0±0	0±0	0±0	0.21±0.01	0.22±0.02	0.24±0.05	0.14±0.02	0.15±0.02	0±0	0.16±0.06	0.06±0.04	0.06±0.04	0.03±0.04	0±0	0±0	0.19±0.04	0.11±0.03	0.06±0.04	0.02±0.02	0±0
	ASV_234	0±0	0±0	0±0	0±0	0±0	0±0	0±0	0±0	0±0	0±0	0±0	0±0	0±0	0.19±0.03	0.12±0.04	0.10±0.02	0±0	0.02±0.03	0±0	0.42±0.09	0.36±0.05	0.32±0.02	0.17±0.01	0.12±0.03
	ASV_261	0.16±0.02	0.12±0.01	0.15±0.01	0.10±0.08	0.12±0.03	0.12±0.01	0±0	0.11±0.01	0.09±0.01	0.12±0.04	0.14±0.04	0.10±0.02	0±0	0.03±0.04	0±0	0.02±0.03	0.05±0.04	0.03±0.04	0±0	0.07±0.01	0.05±0.04	0.02±0.02	0.03±0.03	0.07±0.03
	ASV_280	0.11±0.05	0±0	0±0	0.07±0.05	0.07±0.10	0±0	0±0	0.10±0.01	0.04±0.06	0.09±0.07	0±0	0±0	0±0	0.29±0.02	0.15±0.02	0.06±0.01	0.02±0.02	0±0	0±0	0.18±0.03	0.20±0.02	0.10±0.03	0.10±0.05	0.01±0.02
	ASV_360	0±0	0±0	0±0	0±0	0±0	0±0	0±0	0±0	0±0	0.02±0.03	0±0	0±0	0±0	0±0	0.13±0.02	0.13±0.02	0.12±0.03	0.10±0.01	0±0	0.22±0.02	0.20±0.02	0.17±0.03	0.10±0.08	0.08±0.06
	Other ASVs	0.10±0.09	0.09±0.07	0.09±0.11	0.13±0.09	0.27±0.08	0.09±0.03	0.01±0.01	0.19±0.14	0.26±0.12	0.32±0.15	0.15±0.08	0.12±0.11	0.06±0.03	0.36±0.11	0.3±0.2	0.13±0.07	0.17±0.04	0.09±0.07	0±0	0.50±0.05	0.47±0.27	0.29±0.15	0.24±0.04	0.24±0.15
	ASV_423	0±0	0±0	0±0	0.02±0.02	0±0	0.01±0.01	0±0	0±0	0±0	0±0	0±0	0±0	0±0	0.18±0.01	0.15±0.04	0.14±0.03	0.09±0.02	0.02±0.02	0±0	0.21±0.04	0.13±0.02	0.09±0.03	0.05±0.01	0.02±0.03
	ASV_442	0±0	0±0	0.04±0.03	0.02±0.03	0.04±0.03	0.09±0.03	0±0	0.02±0.03	0.01±0.01	0.02±0.03	0.03±0.02	0.07±0.02	0±0	0.11±0.01	0.08±0.02	0.07±0.02	0.06±0.01	0.05±0.04	0±0	0.10±0.02	0.04±0.03	0.07±0.01	0.10±0	0.07±0.01
	ASV_473	0.06±0.04	0.02±0.03	0.04±0.03	0.05±0.04	0.04±0.03	0.03±0.04	0±0	0.03±0.04	0±0	0.03±0.04	0.13±0.01	0±0	0±0	0.03±0.05	0.10±0.08	0.02±0.03	0.03±0.04	0.02±0.03	0±0	0.05±0.04	0.13±0.04	0.11±0.01	0.07±0.06	0±0
	ASV_007	0.66±0.08	0.56±0.05	0.71±0.02	0.59±0.04	0.85±0.06	0.86±0.13	0±0	0.44±0.03	0.46±0.03	0.49±0	0.70±0.12	0.52±0.08	0±0	1.57±0.04	1.52±0.12	1.54±0.04	1.79±0.07	1.47±0.11	0±0	1.98±0.12	1.96±0.09	2.06±0.12	1.99±0.15	1.78±0.19
	ASV_026	0.18±0.02	0.23±0.03	0.23±0.02	0.22±0.03	0.30±0.01	0.30±0.06	0±0	0.17±0.03	0.12±0.03	0.16±0.02	0.23±0.05	0.20±0.01	0±0	0.63±0.04	0.52±0.03	0.57±0.03	0.50±0.06	0.28±0.02	0±0	0.67±0.02	0.57±0.08	0.64±0.02	0.57±0.05	0.42±0.01
Burkholderiaceae	ASV_053	0.21±0.05	0.21±0.04	0.20±0.03	0.14±0.06	0.19±0.01	0.19±0.07	0±0	0.10±0.07	0.16±0.04	0.16±0.01	0.14±0.10	0±0	0±0	0.33±0.02	0.37±0.04	0.41±0.08	0.38±0.03	0.21±0.03	0±0	0.50±0.03	0.42±0.02	0.41±0.06	0.36±0.06	0.30±0.01
	ASV_108	0.05±0.04	0±0	0.04±0.06	0.06±0.04	0.12±0.04	0.26±0.07	0±0	0.03±0.04	0±0	0.02±0.03	0.07±0.10	0.18±0.06	0±0	0.26±0.01	0.28±0.03	0.25±0.01	0.27±0.05	0.25±0.05	0±0	0.29±0.01	0.29±0.03	0.31±0.03	0.30±0.04	0.27±0.05
	ASV_207	0.03±0.04	0±0	0±0	0.05±0.04	0.07±0.05	0±0	0±0	0±0	0±0	0±0	0.03±0.04	0.02±0.03	0±0	0.17±0.03	0.20±0.01	0.18±0.03	0.23±0.03	0.16±0.04	0±0	0.17±0.03	0.19±0.04	0.21±0.05	0.19±0.01	0.19±0.01
	ASV_218	0.11±0.02	0.03±0.04	0.05±0.06	0.05±0.04	0.12±0.04	0.04±0.05	0±0	0.06±0.04	0.04±0.03	0±0	0±0	0±0	0.05±0.02	0.18±0.01	0.13±0.02	0.12±0.05	0.12±0.05	0.03±0.04	0±0	0.23±0.03	0.17±0.03	0.23±0.04	0.22±0.04	0.05±0.06
	ASV_143	0.03±0.04	0.02±0.03	0.07±0.06	0.05±0.04	0.11±0.01	0.70±0.11	0±0	0±0	0.02±0.03	0±0	0±0	0.36±0.01	0±0	0.20±0.03	0.05±0.07	0.11±0.05	0.12±0.03	0.26±0.03	0±0	0.11±0.03	0.05±0.07	0.10±0.03	0.15±0.02	0.39±0.06
	ASV_337	0±0	0.04±0.03	0.04±0.03	0.01±0.02	0.07±0.02	0.11±0.02	0±0	0.03±0.02	0.02±0.02	0.03±0.03	0.02±0.03	0.09±0.02	0±0	0.09±0.02	0.09±0.06	0.05±0.04	0.14±0.02	0.07±0.02	0±0	0.08±0.01	0.09±0.01	0.09±0.02	0.13±0.01	0.10±0.02
	Other ASVs	0.08±0.01	0.04±0.03	0.15±0.11	0.07±0.10	0.12±0.02	0.42±0.10	0±0	0.07±0.02	0.07±0.05	0.06±0.04	0.10±0.07	0.11±0.03	0.11±0.03	0.36±0.22	0.30±0.12	0.34±0.14	0.27±0.06	0.19±0.07	0±0.01	0.34±0.08	0.26±0.04	0.24±0.05	0.2±0.08	0.28±0.03

Highlight colors indicate as follows: dark red ≥ 1.00%, 0.75% ≤ red < 1.0%, 0.50% ≤ yellow < 0.75%, 0.25% ≤ blue < 0.50%, and 0.10% ≤ green < 0.25%.

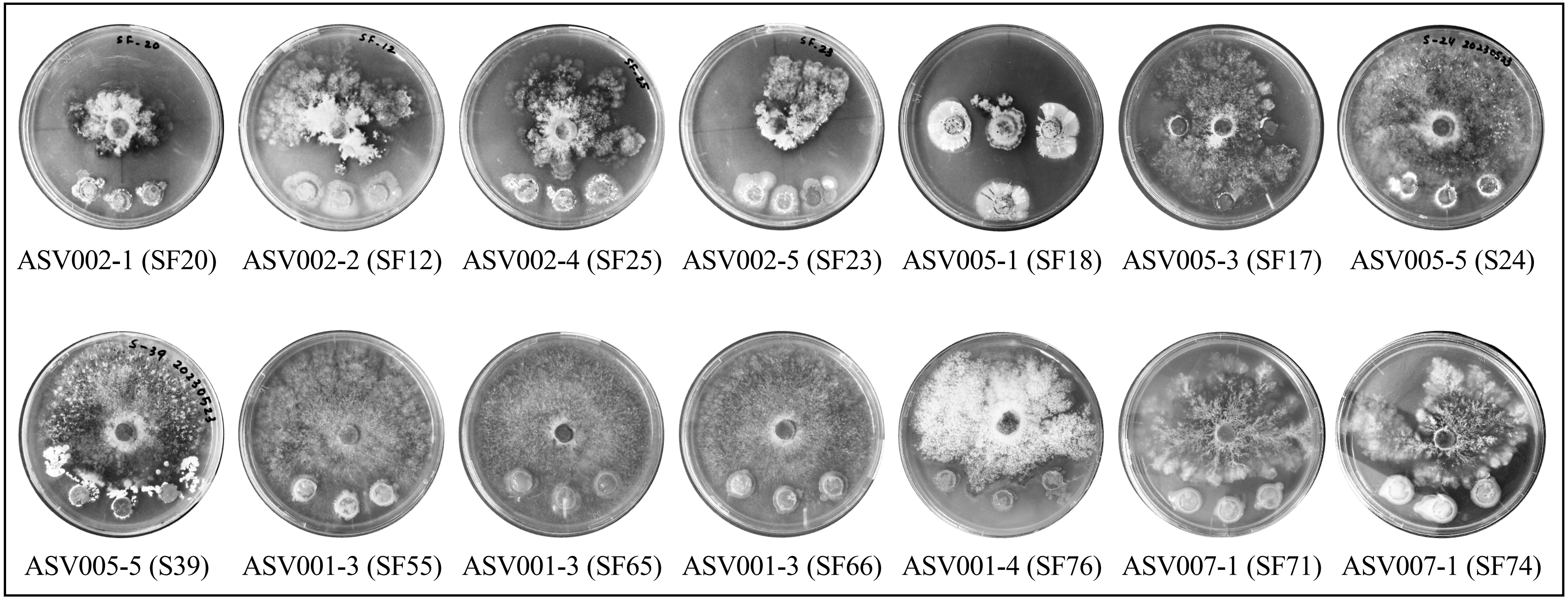

Supplementary Fig. S1. Photographs, other than shown in Fig. 7 of confrontation cultures, which isolates from respective groups and *Diaporthe destruens* were co–cultivated during their development.

The mycelium of *Diaporthe destruens* was placed in the center of agar plates, and the colonies of isolates were placed 1 to 3 cm away from the pathogen.
